# Audit of vancomycin and gentamicin dosing and monitoring in Connolly Hospital Blanchardstown (CHB) 2014

**DOI:** 10.1186/1753-6561-9-S7-A21

**Published:** 2015-10-27

**Authors:** Laura Armstrong, Eoghan O'Neill, Bernie Love

**Affiliations:** 1 Royal College of Surgeons in Ireland, Dublin, Ireland; 2 Department of Microbiology, Connolly Hospital Blanchardstown, Dublin, Ireland; 3 Department of Clinical Microbiology, Royal College of Surgeons in Ireland, Dublin, Ireland; 4 Department of Pharmacy, Connolly Hospital Blanchardstown, Dublin, Ireland

## Background

The dosing of vancomycin and gentamicin relies largely on the weight and renal function of a patient. Due to their narrow therapeutic index, these drugs also require therapeutic drug monitoring (TDM) during treatment to ensure efficacy, reduce potential for oto- and nephro- toxicity, and reduce potential for resistance. Guidelines for the use of vancomycin and gentamicin, as well as a dosing calculator, is in place in CHB. This audit was performed around the appropriate prescribing and monitoring of these two antibiotics in CHB and findings were compared to similar audits carried out in 2010 and 2012.

## Methods

The audit was conducted over two weeks in CHB in June 2014. Patients receiving vancomycin and/or gentamicin were identified from TDM ‘levels’ that had been requested in the Biochemistry Laboratory, CHB. Clinical indication, initial dosing, point in therapy at which first level was taken, and action taken on first level were examined for both drugs.

## Results

A total of 20 patients were audited, 10 patients prescribed vancomycin and 10 patients prescribed gentamicin. 100% of patients were prescribed vancomycin for an appropriate indication and 90% of patients were prescribed gentamicin for an appropriate indication. Appropriate initial dose (based on creatinine clearance and dosing calculator) for vancomycin and gentamicin was 40% and 30% respectively. Checking of the first serum level at the correct point in therapy was 30% for both vancomycin and gentamicin; whilst appropriate action based on this first level was 78% for vancomycin and 66% for gentamicin.

## Conclusions

Although the number of patients included in the audit is small, further improvements in dosing and monitoring of patients prescribed vancomycin and gentamicin in CHB are required. Planned interventions include further education sessions to increase awareness of the dosing calculator and demonstration of its use, revision of the dosing algorithms in the 2015 CHB antibiotic guidelines, and development of a mobile phone application for calculating initial dose requirements.

